# Enzymatic Preparation of Bioactive Peptides Exhibiting ACE Inhibitory Activity from Soybean and Velvet Bean: A Systematic Review

**DOI:** 10.3390/molecules26133822

**Published:** 2021-06-23

**Authors:** Azis Boing Sitanggang, Jessica Eka Putri, Nurheni Sri Palupi, Emmanuel Hatzakis, Elvira Syamsir, Slamet Budijanto

**Affiliations:** 1 Department of Food Science and Technology, Kampus IPB Darmaga, IPB University, Bogor 16680, Indonesia; jessicaep612@gmail.com (J.E.P.); hnpalupi@yahoo.com (N.S.P.); elvira.syamsir@gmail.com (E.S.); slametbu@apps.ipb.ac.id (S.B.); 2 Department of Food Science and Technology, The Ohio State University, 2015 Fyffe Rd, Columbus, OH 43210, USA; chatzakis.1@osu.edu

**Keywords:** angiotensin-I-converting enzyme (ACE), bioactive peptide, endopeptidase, enzymatic hydrolysis, exopeptidase, soybean, velvet bean

## Abstract

The Angiotensin-I-converting enzyme (ACE) is a peptidase with a significant role in the regulation of blood pressure. Within this work, a systematic review on the enzymatic preparation of Angiotensin-I-Converting Enzyme inhibitory (ACEi) peptides is presented. The systematic review is conducted by following PRISMA guidelines. Soybeans and velvet beans are known to have high protein contents that make them suitable as sources of parent proteins for the production of ACEi peptides. Endopeptidase is commonly used in the preparation of soybean-based ACEi peptides, whereas for velvet bean, a combination of both endo- and exopeptidase is frequently used. Soybean glycinin is the preferred substrate for the preparation of ACEi peptides. It contains proline as one of its major amino acids, which exhibits a potent significance in inhibiting ACE. The best enzymatic treatments for producing ACEi peptides from soybean are as follows: proteolytic activity by Protease P (Amano-P from *Aspergillus* sp.), a temperature of 37 °C, a reaction time of 18 h, pH 8.2, and an E/S ratio of 2%. On the other hand, the best enzymatic conditions for producing peptide hydrolysates with high ACEi activity are through sequential hydrolytic activity by the combination of pepsin-pancreatic, an E/S ratio for each enzyme is 10%, the temperature and reaction time for each proteolysis are 37 °C and 0.74 h, respectively, pH for pepsin is 2.0, whereas for pancreatin it is 7.0. As an underutilized pulse, the studies on the enzymatic hydrolysis of velvet bean proteins in producing ACEi peptides are limited. Conclusively, the activity of soybean-based ACEi peptides is found to depend on their molecular sizes, the amino acid residues, and positions. Hydrophobic amino acids with nonpolar side chains, positively charged, branched, and cyclic or aromatic residues are generally preferred for ACEi peptides.

## 1. Introduction

Hypertension is a high prevalence disease and is considered one of the major health problems globally [1]. Lim et al. [2] reported that cardiovascular diseases due to complications of hypertension account for 9.4 million deaths every year. It is therefore of importance to take the appropriate mitigations to reduce the mortality rate due to hypertension. Otherwise known as high blood pressure, hypertension is a medical condition where the arterial blood pressure (BP) is abnormally high. According to the 2019 ACC/AHA Guideline on the Primary Prevention of Cardiovascular Disease [3], a normal BP is described as having a systolic and diastolic pressure of less than 120 and 80 mmHg, respectively (BP < 120/80 mmHg). There are two stages of hypertension. Stage 1 is defined with BP 130–139/80–89 mmHg, while stage 2 hypertension is for BP ≥ 140/90 mm Hg [3]. As mentioned above, hypertension could lead to cardiovascular diseases and stroke. Hypertension is usually treated with blood pressure regulating drugs such as angiotensin-I-converting enzyme (ACE) inhibitors (e.g., lisinopril, captopril), vasodilator, etc. Given the side effects of synthetic ACE inhibitors (e.g., taste disturbances, cough, and swelling of the lower layer of human skin or angioneurotic edema) [4], various investigations have been afforded to find potent ACE inhibitors from natural products, especially from food proteins. As a result of the increasing interest regarding functional foods in the past few years, it has been reported that food proteins-derived bioactive peptides have several benevolent effects on human health, including inhibitory activity against ACE [5,6]. Therefore, bioactive peptides can be considered as an alternative for managing hypertension.

A bioactive peptide is defined as an organic compound with a positive impact on human health (e.g., inhibitory activity against ACE, antioxidant capacity, antimicrobial activity, anti-thrombotic, immunomodulatory, etc.) which consists of 2–20 amino acids joined by covalent bonds called peptide bonds [7]. In the digestive system of the human body, bioactive peptides are liberated by digestive proteases, such as pepsin or microbial enzymes. Additionally, processing food and ripening can release bioactive peptides.

Nature is an abundant source of bioactive peptides produced by organisms such as plants and animals. Although animal products remain the greatest source of bioactive peptides, this work will mainly discuss plant source bioactive peptides derived specifically from soybean and velvet beans. Soybean nutritional content consists of 35–40% protein, 20% lipids, and 9% dietary fiber based on dry-weight soybean [8,9]. Because of its high protein content, soybean is mostly utilized as a source of bioactive peptides among other plants. Meanwhile, a less well-known type of legumes called velvet bean has a nutritional content of approximately 25% protein and 14% crude fat based on its dry weight [10]. As both beans are considered as potent protein sources in the human diet, their utilization as sources of parent proteins for producing bioactive peptides is promising. However, in the case of velvet bean, studies related to its utilization as a parent protein source are scarce. Thus, it is important to elucidate the technological approach of producing velvet bean-derived peptides especially for inhibiting ACE activity.

Highlighting the elevating interest in the bioactive peptides as part of the diet and the higher prevalence of the non-communicable disease of hypertension, this systematic review discusses the advances in the enzymatic preparation of bioactive peptides from soybean and velvet beans exhibiting Angiotensin-I-Converting Enzyme inhibitory (ACEi) activity. This study focuses its discussion on the optimum hydrolytic conditions required to produce ACEi peptides and the influence of peptides’ intrinsic properties (i.e., amino acid residues and their arrangement in the sequence, molecular weight, hydrophobicity) on the ACEi activity.

## 2. Results

### 2.1. Substrate Preparation as Source of ACEi Peptides from Soybean and Velvet Bean

The preparation of substrates from soybeans is rarely discussed in the literature. Substrates from soybeans as sources of parent proteins can be soy protein concentrate or isolate, soybean flour-rich in protein, and principal soybean storage proteins (i.e., glycinin or β-conglycinin). Gouda et al. [11] prepared the soy protein substrate, glycinin. This method follows a previously described method developed in a study by Rao and Rao [12] with the use of (NH_4_)_2_SO_4_ precipitation and centrifugation. Water containing β-mercaptoethanol (0.1% *v*/*v*) is used to extract defatted soybean flour for 4–6 h under constant agitation. The solution is then centrifuged at 6000–8000 rpm for 45 min at 25 °C, followed by the addition of dry MgCl_2_ until the final MgCl_2_ concentration in the solution reaches 5 mM. Glycinin is collected by centrifugation, and the precipitate is dried with a freeze drier. Freeze drying is used as a preferred water removal method because it has the advantage to cause less damage to the structure of the protein substrate. Nevertheless, the fractionation of glycinin in most studies involves the precipitation of the alkaline soy protein extract at pH 6.3–7.0 [13,14,15].

For the preparation of the velvet bean substrate, wet fractionation is the method that is commonly used [16,17,18]. Initially, velvet bean flour is prepared by grounding the grains with a disk mill followed by sieving. The prepared bean flour then undergoes suspension in 3% sodium bisulfite with a 1:6 ratio (*w*:*v*) and left to soak for an hour with a constant agitation under alkaline pH (pH = 8). The role of sodium bisulfite is to increase the solubility of the velvet bean protein. Abtahi and Aminlari [19] stated that the modification of protein with a chemical treatment, such as sodium bisulfite, increases the protein dispersibility index (PDI). After fiber solid separation and washing with 3% sodium bisulfite, the protein-starch suspension is then left to sediment for 30 min. The purpose of sedimentation is to recover starch. The pH of protein solution pH is adjusted to an isoelectric point (i.e., pH 4.2) using 1.0 M HCl solution. The precipitate is obtained by centrifuging the solution at 1317× *g* for 20 min and further dried using a freeze-drier at −47 °C and pressure of 13 × 10^−3^ mbar [16,17,18]. In another study by Mugendi et al. [20] who characterized the nutritional properties of velvet bean protein isolate, the extraction was conducted with distilled water at pH 9 followed by centrifugation. The pH of the extract was then adjusted to 4.5 to precipitate the protein.

### 2.2. Hydrolytic Conditions for Producing ACEi Peptides from Soybean and Velvet Bean Protein Substrates

Enzymes for proteolysis are classified as endopeptidases and exopeptidases, based on the site of action on the substrate. Exopeptidases hydrolyze at the N- or C-terminal ends of the peptide, while endopeptidases cleave peptide bonds within and distant from the ends of a polypeptide chain or at the non-terminals of the sequence [21]. The most common enzymes used for producing soybean-based bioactive peptides are pepsin [22,23], papain [6,24], alcalase [25,26,27], proteinase from M. purpureus [28], trypsin, chymotrypsin, ginger protease, and Amano Protease from *Aspergillus* sp. [11], and protease D3 from *E. coli* strain JM109 [29]. All of these enzymes are endopeptidases. Endopeptidases, such as alcalase and proteinase K produce short-chain hydrophobic amino acids which are preferred in enhancing ACEi activity [21]. Additionally, prolyl endopeptidases such as Protease P from *Aspergillus niger* are often used as it can yield in proline-containing bioactive peptides which are favored for their strong affinity to ACE [30]. Hydrolytic conditions of soybean proteins for producing ACEi peptides are shown in Table 1.

For velvet bean, the proteolytic enzymes reported limitedly in the literature are a combination of pepsin-pancreatin [16,17,18] and alcalase–flavourzyme [16,17]. In contrast to soybean-derived peptides, for velvet bean sourced peptides, the hydrolysis is conducted with a combination of both endopeptidase and exopeptidase. The application of both endo- and exo-peptidase allows it to have a broad cleavage action and produce a shorter chain of peptides. Table 2 shows the enzymatic hydrolysis conditions of velvet bean-derived proteins.

## 3. Discussion

### 3.1. Hydrolytic Conditions of Soybean-Based Bioactive Peptides Preparation

Besides fermentation, bioactive peptides can also be produced by the hydrolytic activity of proteases on soybean parent proteins [24]. Important factors to consider in producing soybean-derived bioactive peptides through proteolysis are the type of enzyme, reaction temperature, time of hydrolysis, pH, and enzyme-substrate ratio (E/S) [32]. At a low enzyme-to-substrate (E/S) ratio, the enzyme will continuously cut the most susceptible peptide bonds during the hydrolysis period. Meanwhile, with the increase of E/S ratio, cleavage action is faster during the initial stage of hydrolysis and becomes slower at a later stage. This is because at the initial stage, the reaction is spent by rapid cleavage of the susceptible peptide bonds and at a later stage, enzymes degrade the less susceptible peptide bonds [33]. The reported E/S ratios for producing ACEi peptides from soybean proteins are mostly less than 10%, with majority at 4 and 6%.

Among others, common proteolytic enzymes used for the production of bioactive peptides from soybeans include pepsin, alcalase, and protease D3. Pepsin is a protease that hydrolyzes peptide bonds between the aromatic amino acids such as phenylalanine, tryptophan, and tyrosine [34]. It is classified as an endopeptidase. Wang et al. [35] stated that pepsin effectively degrades proteins between pH 1.2–2.5 and the optimal pH for pepsin’s proteolytic activity is 1.6 with a temperature of 37 °C, and an E/S ratio of 10 U:1 μg. According to Chen et al. [22,23], the optimum pH used for pepsin hydrolysis was 2, while for Lo and Li-Chan [31], the optimum pH used was 5. This discrepancy is likely influenced by the unique physiochemical properties of each of the substrate proteins in different pH environments. Chen et al. [22] selected a hydrolysis temperature of 37 °C for 24 h while in the other study by the same authors [23], 39 °C was used with half the time from the prior study.

Alcalase is known to produce peptides with hydrophobic domains at the C-terminal with optimum conditions of hydrolysis as follows: 56 °C in temperature, pH 7, E/S ratio of 2% (*v*/*w*) for 6 h [36]. Wu and Ding [25], Rayaprolu et al. [26], Li et al. [27] used alcalase in producing bioactive peptides from soybean with different conditions. Both Wu & Ding [25] and Rayaprolu et al. [26] used 50–55 °C hydrolysis temperatures, which complies with the optimal hydrolyzing temperature for alcalase for producing other types of bioactive peptides [37]. Meanwhile, Li et al. [27] used reaction temperature of 37 °C. As for the hydrolysis time, it varies in all three studies with Wu & Ding [25] reporting 12 h, Rayaprolu et al. [26] 1 h and Li et al. [27] 0.25 h. All three used alkaline hydrolytic conditions as alcalase might have optimum pH up to 10 [37].

The conditions for hydrolyzing soybean proteins for producing ACEi peptides may be different from the conditions where the optimum catalytic activity of the enzyme appears using standard substrate (casein, albumin, etc.). A study conducted by Yasuda et al. [38] suggested that protease derived from *Monascus prureus* has an optimum temperature of 50 °C and a pH of 3.2 to achieve optimum activity. However, utilizing the same enzyme source, Kuba et al. [28] conducted the hydrolysis at 37 °C with a pH of 3.3 for producing ACEi peptides.

Gouda et al. [11] compared four different enzymes for the production of bioactive peptides derived from soybeans, namely trypsin, chymotrypsin, protease P, and ginger protease. Trypsin and chymotrypsin used in that study were bovine-sourced and in both cases, the hydrolysis conditions were 37 °C at a pH of 8.2 for 18 h. These enzymes are digestive enzymes found in the small intestine which has a pH of 8–9. Hydrolysis of β-Lactoglobulin with trypsin that was reported in another study [39], carried out at pH 7.8, 37 °C, and a hydrolysis time of 2.42 h. Meanwhile, Kimball et al. [40] hydrolysed soybean proteins using chymotrypsin with optimum conditions as follows: E/S ratio of 2/100 (*w*/*w*) at 37 °C using a reaction time of 20–30 min. Reaction with protease P has also been used with the same hydrolysis conditions as trypsin and chymotrypsin. Siala et al. [41] reported that for protease derived from *Aspergillus niger,* the optimum pH was 4.0, while the enzyme was highly active at a temperature range of 30–60 °C, with an optimum activity at 50 °C. Meanwhile, ginger sourced protease, a cysteine protease indicated by the presence of cysteine residual at the active site of the enzyme, is best utilized at a pH range of 6–8 with a temperature of 60 °C [42]. Protease D3 is another novel cysteine peptidase, purified from germinating soybean cotyledon. According to Miwa [43], protease D3 worked at an optimum temperature of 40 °C with a pH above 4. Kodera and Nio [29] confirmed the former statement by utilizing protease D3 at 37–40 °C, at a pH of 4.5.

The optimum pH, hydrolysis time, E/S ratio, and reacting temperature used to produce ACEi peptides from soybean-derived proteins vary depending on the enzyme utilized. However, the optimum pH and temperature are relatively consistent, at least for pepsin and alcalase. Pepsin is optimum at pHs 1.2–2.5 and temperatures of 37–39 °C, whereas for alcalase pHs 7–9 and temperatures of 50–60 °C are the used conditions for producing ACEi peptides from soybean proteins.

### 3.2. Hydrolytic Conditions of Velvet Bean-Based Bioactive Peptide Preparation

Hydrolysis of parent proteins from velvet bean adopts the combination of endopeptidase and exopeptidase enzymes. Chalé et al. [17] and Segura-Campos et al. [16] both conducted hydrolysis of velvet bean concentrate using a pepsin-pancreatin combination and an alcalase-flavourzyme combination, while Tuz and Campos [18] used a combination of pepsin–pancreatin. The hydrolytic conditions for pepsin and pancreatin hydrolysis were 37 °C for 15 min at pH 2 for pepsin and at pH 7 for pancreatin. This complies with the previous study by Wang et al. [35] that utilizes pepsin for degrading both pepsin susceptible and resistant proteins. Meanwhile, flavourzyme, a mixture of endo and exopeptidases found in *Aspergillus oryzae*, works actively in the temperature of 50–55 °C and pH 5–7. Chalé et al. [17] and Segura-Campos et al. [16] used flavourzyme in combination with alcalase to hydrolyze velvet bean-derived proteins with the same hydrolysis condition. The optimum conditions used were 50 °C, pH 7 for 15 min. These conditions match Nguyen et al. [44] optimum conditions, however, in hydrolysing soybean protein. The range of the ACE inhibition activities (half maximal inhibitory concentration/IC_50_) from velvet bean peptide fraction is 0.0009–10.2 μg/mL [17,18]. The best enzymatic treatments having ACE IC_50_ of 0.0009 μg/mL as follows: the sequential hydrolytic activity by the combination of pepsin–pancreatin, E/S ratio for each is 10%, the temperature and reaction time for each is 37 °C and 0.74 h, respectively, and pH for pepsin and pancreatin is 2 and 7, respectively. Unfortunately, there is no study reported to identify the structures of ACEi peptides from velvet bean proteins at the time this work is caried out. Therefore, the following discussion focuses on ACEi peptides derived from soybean proteins.

### 3.3. Bioactive Peptides Exhibiting ACEi Activity from Soybean Proteins

The soybean protein isolate consists of various proteins which are grouped into four main protein classes, namely 2S (albumin), 7S (β-conglycinin), 11S (glycinin), and 15S. These storage proteins are grouped based on the sedimentation coefficients when the protein solution is subjected to a centrifugal field [45]. Although there are other minor proteins in soybeans, such as hemagglutinins, trypsin inhibitor, and intrinsic enzymes, 7S and 11S proteins are the most abundant and account for 75% of total storage protein content [9,46].

For 11S, there are five identified protein subunits which are divided into two groups based on homology. Group 1 comprises of G1 (53.6 kDa), G2 (52.4 kDa), G3 (52.2 kDa), and Group 2 with G4 (61.2 kDa) and G5 (55.4 kDa) [47]. β-conglycinin is the major glycoprotein for 7S storage protein. It consists of three major subunits, namely α (ca. 67 kDa), α′ (ca. 71 kDa), and β (ca. 50 kDa), in which all three have different physiochemical properties. The 7S fraction of globulins also comprises two more proteins, in addition to β-conglycinin, namely γ-conglycinin and B_o_-conglycinin. In total, these soybean storage proteins are considered as potent parent proteins for producing ACEi peptides. In Table 3, the identified ACEi peptides from published literature are presented. Additionally, these identified ACEi peptides are matched with amino acid sequences from the typical soybean parent proteins, such as glycinin, β-conglycinin, and 2S albumin, to elucidate the source of those identified ACEi peptides.

Chen et al. [22] identified five peptides (i.e., IA, TLAGAG, PPL, ITLL, and VMALPG) exhibiting ACEi activity produced from soybean proteins. Four other ACEi peptides were isolated from β-conglycinin and glycinin, namely LAIPVNKP, LPHF, SPYP, and WL by Kuba et al. [28]. Moreover, Gouda et al. [11] isolated VLIVP from glycinin. Other ACEi peptides isolated from soybeans were YVVFK, PNNKPFQ, NWGPLV, and IPPGVPYWT [29]. A comparison of those identified ACEi peptides with the SWISS-PROT database of soybean-derived parent proteins shows that most of the identified ACEi peptides are found in soybean protein, G1-G2, G4, α, α′, β, and 2S albumin. Identified ACEi peptides that are consistent with the database are IA, NWGPLV, SPYP, WL, LPHF, LAIPVNKP, VLIVP, and PNNKPFQ (refer to Table 3).

**Figure 1:**
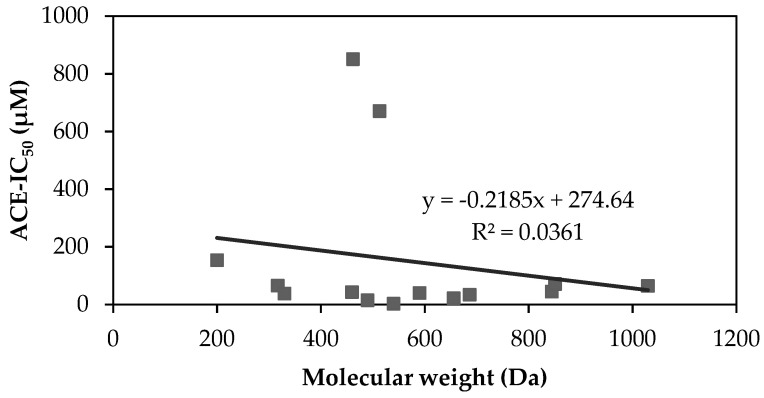
Correlation between ACEi activity and peptide’s molecular weight. MWs and IC_50_ values are taken from Table 4.

The molecular weight (MW) of an ACEi peptide can determine its affinity with ACE as the binding site might be too narrow for large MW peptides. ACEi activities from the corresponding identified peptides in Table 3 are evaluated and VLIVP, a peptide with a MW of 540 Da, is found to have the highest ACEi activity with an IC_50_ of 1.69 µM (Table 4). On the contrary, the lowest ACEi activity belongs to SPYP with an IC_50_ value of 850 µM. Despite having a considerably low MW (i.e., 462 Da), SPYP has the lowest ACEi activity. A correlation between MWs of ACEi peptides and their inhibition values is shown in Figure 1. The coefficient determination is 0.036, which indicates a very weak correlation. This suggests that ACEi activity does not entirely depend on peptide’s MW. Especially when MW of peptide is lower than 1 kDa, the molecular structure of ACEi peptide remarkably dictates the interaction between an ACEi peptide and ACE’s active side. These structural factors, such as peptide hydrophobicity and the types of amino acid residues encrypted within peptide strand are reported to influence the inhibition activity on ACE [24,48,49].

Peptides with strong ACEi activities are mostly composed of hydrophobic amino acids with nonpolar side chain, positively charged, branched, and cyclic or aromatic residues, and proline at C terminus [24,49,50]. ACE contains HEXXH (i.e., histidine, glutamic acid, unknown, unknown, and histidine) as the active site, where two histidines (His_383_ and His_387_), together with the glutamate (Glu_411_), form zinc (Zn^2+^) binding ligands [51]. According to Jimsheena and Gowda [52], the presence of proline at the C-terminus in QRP and its short coordination distance, especially the carbonyl oxygen of the peptide bond between Q and R (3.2 Å) led to an increase in ACE inhibition. Bechaux et al. [53] and Aluko [49] also stated that the preferred ACEi peptide has an N-terminal branched-chain amino acid (aliphatic side chain with a branch), and of the C-terminal proline, aromatic, branched, or basic amino acids. Thus, peptides with N terminal of V or I, and C terminal of W, Y, P, and F are more preferred. The presence of proline in peptide sequences exhibiting ACEi activity is also reported by Sitanggang et al. [24] on fermented soybean (tempeh)-derived peptides (i.e., NEGDVLVIPPGVP, APIDVVVPPGNT, VAPTPNVPPYAG, FLVPPQ, FLVPPQE). It is known that the existence of proline and hydroxyproline in peptides is unaffected by the action of digestive proteases especially tripeptides with C-terminal proline-proline [54]. The resistance of bioactive peptides from the gastrointestinal proteases might be beneficial in maintaining the activity of ACEi peptides.

The fact that VLIVP has the highest ACEi activity (see Table 4) is consistent with the former reasoning statements [24,49,52,53]. VLIVP has a branched amino acid, namely valine at the N-terminal site and proline at C-terminal. Another factor that also contributes to the lowest IC_50_ value is the use of glycinin as a substrate for proteolysis [11]. According to Riblett et al. [55], glycinin contained proline as one of its major amino acids, which was a preferred amino acid in producing ACEi peptides, compared to β-conglycinin. Considering that VLIVP has the highest ACEi activity, the concluded best enzymatic treatments for producing ACEi peptides from soybean parent proteins as follows: proteolytic activity by protease P (Amano-P from *Aspergillus* sp.), the temperature of 37 °C, a reaction time of 18 h, pH 8.2 and E/S ratio of 2% [11].

## 4. Materials and Methods

The approach in constructing this review followed Carey et al. [56] which is a step-by-step guideline in conducting a systematic review. General views of the procedures consist of initial planning, conducting searches, data extraction, and quality analysis.

### 4.1. Defining a Research Question, Inclusion and Exclusion of Articles

This work carried out a literature review to search for relevant references. As a start, a review question was made. The review question chosen to conduct the research was “What are the best enzymatic treatments for producing potent bioactive peptides exhibiting ACE inhibitory activities from soybean and velvet bean proteins?” This scientific question was chosen to better clarify the purposes of this review, which were to demonstrate the optimum hydrolytic conditions that produce a high ACEi activity of a bioactive peptide and to evaluate the influence of structural factors of the identified bioactive peptide(s) on the ACEi activity. Based on that, the review question was categorized into a search tool, namely PEO, which was used to organize framework of main concepts [57]. PEO stands for population, exposure, and outcome. Within this study, the parent proteins from soybean and velvet bean, the enzymatic preparation conditions, and bioactive peptides exhibiting ACEi activity were considered as population, exposure, and outcome, respectively. 

Furthermore, the studies or articles included were selected based on several criteria that were considered important for reference selection. Firstly, only studies published in English were included. There were no limitations regarding the publication dates. In addition, studies unrelated to the enzymatic preparations of velvet bean or soybean bioactive peptides exhibiting the ACEi effect were excluded.

### 4.2. Conducting and Reviewing the Search

**Figure 2:**
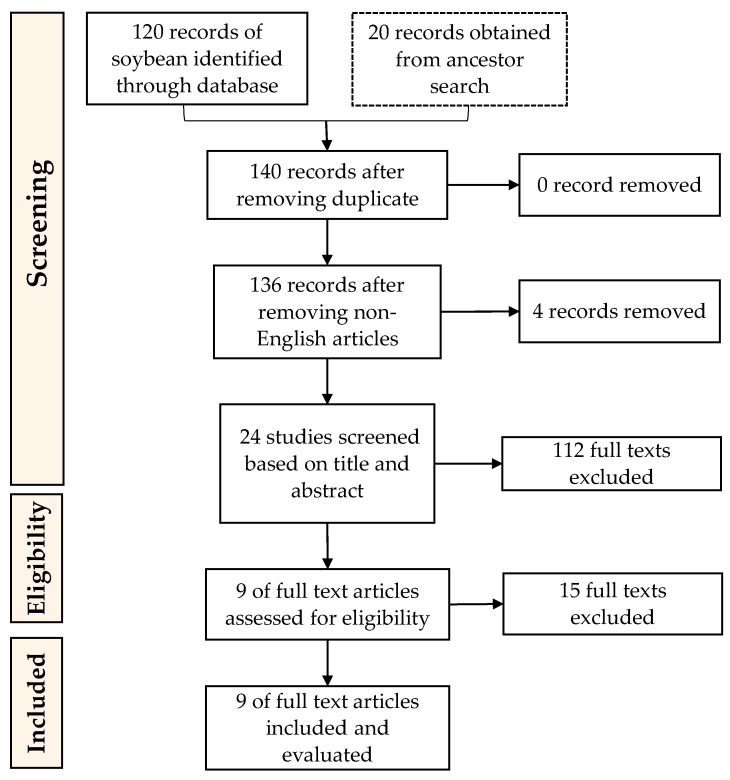
PRISMA guidelines for the inclusion of articles on the enzymatic preparation of ACEi peptides from soybean proteins.

**Figure 3:**
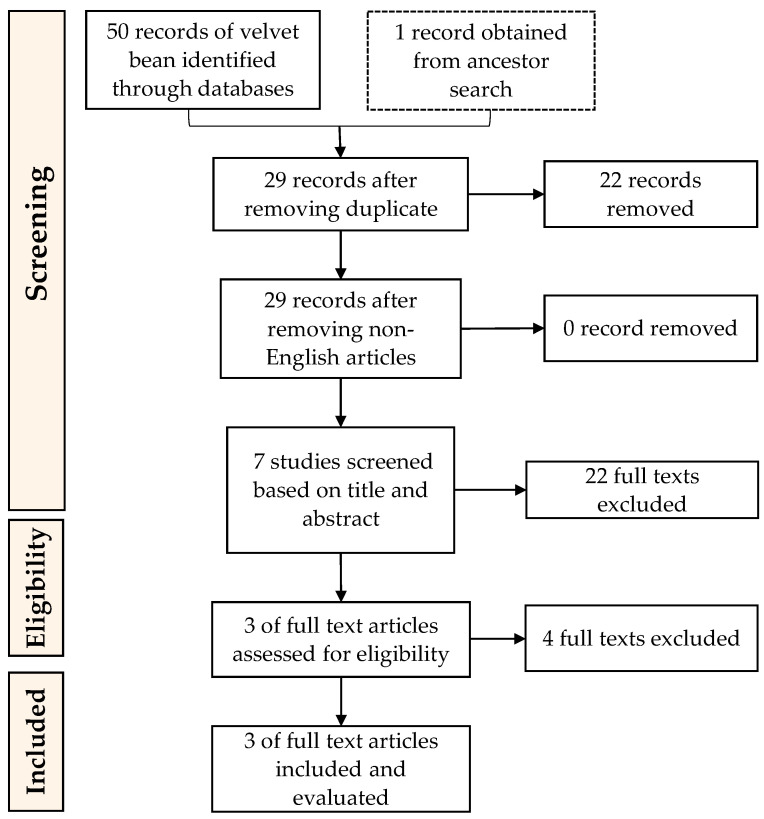
PRISMA guidelines for the inclusion of articles on the enzymatic preparation of ACEi peptides from velvet bean proteins.

References corresponding to this work were selected using “Preferred Reporting Items for Systematic reviews and Meta-Analysis” (PRISMA) guidelines (Figure 2 and Figure 3) as it is considered as the common guideline for conducting systematic reviews [58]. The following steps used in conducting this research were (1) Data collection (conducting searches of articles relevant to this work in database); (2) Data screening (selecting the articles based on criteria or classification determined beforehand); (3) Data integration (integrating selected references found and making the selection); (4) Data analysis (analysing the integrated data); (5) Data conclusion (providing results of the review).

Studies relevant to the review were searched using databases such as Google Scholar (https://scholar.google.co.id; accessed on 30 May 2020), and Wiley (https://onlinelibrary.wiley.com; accessed on 30 May 2020). These databases were chosen as they are considered as common database used that support the Boolean search. Boolean search is a structured search where users can include several operations (AND, OR, NOT) to specify or broaden the search results. In searching data or references related to the ACEi peptides from soybeans, the Boolean used was “enzymatic preparation” AND “bioactive peptide” AND soybean AND (“blood pressure” OR hypertension). For velvet bean, the Boolean used was “bioactive peptide” AND (*Mucuna* OR “velvet bean”) AND (“blood pressure” OR hypertension).

Records of references related to the research topic of soybean ACEi peptides resulted in a total of 120 records from database searches and 20 records from ancient searches. Ancestor searches were also used to add more corresponding references to this study. Ancestor searches are conducted by going through the references from the selected articles to obtain more data sources [59]. Management and tracking of the records were by Mendeley (Mendeley Ltd., Elsevier, Amsterdam, The Netherlands), a reference manager that helps to collect references and organize citations. Duplicates were not found, although there were four non-English articles. References from both databases and ancestor searches were screened based on the inclusion and exclusion described above. After a further screening process, nine soybean records were used and evaluated in this research. The last screening process was to remove texts that are not specifically about soybean or not specifically discussed ACEi activity. As for velvet beans, a total of 50 records from database searches and one record from ancient searches were found. After merging duplicates and removing the non-English articles, 56 records were left to be screened based on the conformity of the title and abstract, and the research topic. The texts excluded here were studies that are not specifically about velvet bean or did not specifically discuss ACEi activity. This resulted in three velvet bean ACEi peptide-related articles to be included in this review.

## 5. Conclusions

The preparation of ACEi peptides from soybean in most literature is performed by the proteolytic activity of endopeptidases. The best enzymatic treatments for producing ACEi peptides from soybean parent proteins as follows: proteolytic activity by Protease P (Amano-P from *Aspergillus* sp.), a temperature of 37 °C, a reaction time of 18 h, pH 8.2 and an E/S ratio of 2%. The identified ACEi peptide having an IC_50_ of 1.69 µM is VLIVP. This peptide has a relatively low MW of 450 Da, which is presumably important to have it buried in the active site of ACE. Most importantly, this peptide has V and P at N- and C-terminal, respectively, which is a preferred configuration for enhancing ACEi activity. It is worth mentioning that besides MW of an ACEi peptide, other structural factors, such as peptide hydrophobicity and the types of amino acid residues encrypted within peptide strand, are also influential to enhance the ACEi activity.

As for velvet bean, the enzymes used for the hydrolytic actions are a combination of exo- and endo-peptidase. The best enzymatic treatments for producing peptide hydrolysates with a high ACEi activity are as follows: sequential hydrolytic activity by the combination of pepsin–pancreatic, an E/S ratio for each is 10%, the temperature and reaction time for each are 37 °C and 0.74 h, respectively, pH for pepsin is 2, whereas for pancreatin it is 7. Studies on the enzymatic hydrolysis of velvet bean proteins for producing ACEi peptides are limited. Additionally, there are no studies related to the identification of the molecular structures of ACEi peptides. Since velvet bean also has a high protein content, it is thus considered as a potent source of ACEi peptides. Therefore, the research interest should be directed in this area in the future.

## Figures and Tables

**Table 1:** Enzymatic hydrolysis conditions of soybean proteins using endopeptidases to produce ACEi peptides.

Enzyme	Substrate	Temp. (°C)	Time (h)	pH	Enzyme-to-Substrate Ratio E/S	Peptide Sequence	Ref.
Pepsin	Protein concentrate	37	24	2	6%	IA	Chen et al. [22]
						TLAGAG	
						PPL	
						ITLL	
						VMALPG	
Pepsin	Protein isolate	39	12	2	3%	-	Chen et al. [23]
	Acid-precipitated protein						
Alcalase	Protein concentrate	50	12	9	4%	-	Wu & Ding [25]
*M. purpureus* acid proteinase	β-conglycinin	37	10	3.3	-	LAIPVNKP	Kuba et al. [28]
						LPHF	
	Glycinin					SPYP	
						WL	
Bovine trypsin	Glycinin	37	18	8.2	2%	VLIVP	Gouda et al. [11]
Bovine chymotrypsin		37	18	8.2			
Ginger protease		50	16	6			
Protease P (Amano-P from *Aspergillus* sp.)		37	18	8.2			
Protease D3 from *E. coli* strain JM109	Protein isolate	37–40	24–48	4.5	0.2%	YVVFK	Kodera & Nio [29]
						PNNKPFQ	
						NWGPLV	
						IPPGVPYWT	
Pepsin	Protein isolate	37	1	5.3	4%	-	Lo & Li-Chan [31]
Pancreatin		37	2	7.5			
Alcalase	Protein isolate	55	1	8	-	-	Rayaprolu et al. [26]
Alcalase	Protein isolate	30	0.25	9	6%	-	Li et al. [27]

**Table 2:** Enzymatic hydrolysis conditions of velvet bean protein concentrate to produce ACEi peptides.

Enzyme	Enzyme Type	Hydrolysis Conditions				Ref.
		Temp. (°C)	Time (h)	pH	Enzyme-to-Substrate Ratio E/S	
Pepsin	Endopeptidase	37	0.75	2	10%	Herrera-Chale et al. [17]
Pancreatin	Exopeptidase	37	0.75	7.5		
Alcalase	Endopeptidase	50	0.75	8		
Flavourzyme	Exopeptidase	50	0.75	7		
Pepsin	Endopeptidase	37	0.75	2	10%	Tuz & Campos [18]
Pancreatin	Exopeptidase	37	0.75	7		
Pepsin	Endopeptidase	37	0.75	2	10%	Segura-Campos et al. [16]
Pancreatin	Exopeptidase	37	0.75	7		
Alcalase	Endopeptidase	50	0.75	8		
Flavourzyme	Exopeptidase	50	0.75	7		

**Table 3:** Identified ACEi peptides and their corresponding specific soybean proteins as sources.

Soybean Protein	Total Molecular Weight/MW (Da)		UniProt Entry	Peptide Strand	Location of Peptide Strand from N-Terminal	Identified ACEi Peptide from Literature		
						AA Residues	MW (Da)	Ref.
Glycinin	G1	55,706	P04776	L**IA**VPTGVAW	141–150	IA	202	Chen et al. [22]
				ALS**WL**RLSAE	351–360	WL	317	Kuba et al. [28]
				**VLIVP**QNFVV	411–420	VLIVP	540	Gouda et al. [11]
	G2	54,391	P04405	AL**WL**LKLSAQ	341–350	WL	317	Kuba et al. [28]
				TWN**PNNKPFQ**	51–60	PNNKPFQ	844	Kodera & Nio [29]
	G3				-			
	G4	63,797	P02858	HLPSY**SPYP**R	81–90	SPYP	462	Kuba et al. [28]
				MII**IA**QGKGA	91–100	IA	202	Chen et al. [22]
				SFNTNED**IA**E	241–250	IA	202	Chen et al. [22]
				EN**IA**RPSRAD	391–400	IA	202	Chen et al. [22]
				YEG**NWGPLV**N	541–550	NWGPLV	586	Kodera & Nio [29]
	G5	57,956	P04347	GLE**YVVFK**TH	461–470	YVVFK	655	Kodera & Nio [29]
β–conglycinin	α	70,306	P0DO16	VSFG**IA**YWEK	21–30	IA	202	Chen et al. [22]
				NENLRLIT**LA** **IPVNKP**GRFE	301–320	LAIPVNKP	851	Kuba et al. [28]
				L**LPHF**NSKA	451–460	LPHF	513	Kuba et al. [28]
	α’	72,228	P11827	VSFG**IA**YWEK	21–30	IA	202	Chen et al. [22]
				RMIT**LAIPVN** **KP**GRFESFFL	321–340	LAIPVNKP	851	Kuba et al. [28]
	β	50,476	P25974	QNLKIIK**LAI** **PVNKP**GRYDD	141–160	LAIPVNKP	851	Kuba et al. [28]
				EGALL**LPHF**N	281–290	LPHF	513	Kuba et al. [28]
2S albumin	18,460		P19594	LLFC**IA**HTCS	11–20	IA	202	Chen et al. [22]

**Table 4:** Molecular weights and inhibition values of ACEi bioactive peptides.

No	Sequence	MW (Da)	ACEi IC_50_ (μM)	Ref.
1	IA	200	153	Chen et al. [22]
2	WL	317	65	Kuba et al. [28]
3	PPL	330	37	Chen et al. [22]
4	ITLL	460	42	
5	SPYP	462	850	Kuba et al. [28]
6	TLAGAG	490	14	Chen et al. [22]
7	LPHF	513	670	Kuba et al. [28]
8	VLIVP	540	1.69	Gouda et al. [11]
9	VMALPG	590	39	Chen et al. [22]
10	YVVFK	655.62	21	Kodera & Nio [29]
11	NWGPLV	686.56	33	
12	PNNKPFQ	844.59	44	
13	LAIPVNKP	851	70	Kuba et al. [28]
14	IPPGVPYWT	1029.69	64	Kodera & Nio [29]

## Data Availability

Data sharing not applicable.
